# Highly Luminescent and Scintillating Hybrid Halide of (C_13_H_25_N)_2_[MnBr_4_] Enabled by Rigid Cation

**DOI:** 10.3390/molecules30102157

**Published:** 2025-05-14

**Authors:** Renfu Li, Lulu Jiang, Qinghua Zou, Jianlong Bai, Lingkun Wu, Jianrong Li, Jinsheng Liao

**Affiliations:** Jiangxi Provincial Key Laboratory of Functional Crystalline Materials Chemistry, Jiangxi University of Science and Technology, Ganzhou 341000, China; lirenfu@fjirsm.ac.cn (R.L.); baekrrlulu@163.com (L.J.); zouqinghua1126@163.com (Q.Z.); baijianlong666@gmail.com (J.B.); wlk492599141@163.com (L.W.)

**Keywords:** organic inorganic hybrid, Mn halide, luminescence, scintillator

## Abstract

Organic–inorganic hybrid manganese halides (OIMnHs) have attracted significant attention in the field of optoelectronics due to their outstanding optical properties and low toxicity. However, the development of crystalline compounds with scintillating properties and high light yield remains a significant challenge. In this study, a simple solution method was employed to successfully synthesize a new zero-dimensional (0-D) scintillation crystal, (C_13_H_25_N)_2_[MnBr_4_] (C_13_H_25_N = trimethyladamantan-1-aminium). The introduction of bulky and rigid organic cations not only spatially isolates the [MnBr_4_]^2−^ tetrahedrons but also effectively expands the Mn···Mn distance, thereby suppressing the concentration quenching and self-absorption effects. This structural design achieves a high photoluminescence quantum yield of about 63.8% at room temperature and a remarkable light yield of 44,300 photons MeV^−1^. After multiple irradiation cycles, the material retains its stable radiative characteristics. This work highlights the key role of rigid cation engineering in improving luminescence efficiency and scintillation performance and provides new ideas for designing efficient and nontoxic OIMnH-based scintillators.

## 1. Introduction

Scintillators, as radiation-responsive luminescent materials, are extensively utilized in radiation detection [1,2], medical imaging [3,4], and security applications [5,6]. Over the past few decades, numerous commercial scintillators with exceptional performance have been developed, such as CsI:Tl, Bi_4_Ge_3_O_12_ (BGO), and CdWO_4_ (CWO). However, their complex crystal growth process and high manufacturing cost severely limit their large-scale application. Organic–inorganic hybrid metal halides (OIMHs) have important application potential in the optoelectronic field (lighting, anti-counterfeiting, display technology) as well as radiation detection and bioimaging due to their excellent optoelectronic properties and tunable structure [7,8,9,10,11,12,13,14]. Although lead-based perovskites have high carrier mobility and strong X-ray absorption capabilities, their lead toxicity restricts their applications. Manganese-based materials have become candidates for new optical scintillators due to their low cost, weak self-absorption, and excellent optical properties [15,16,17]. Organic–inorganic hybrid manganese halides (OIMnHs) not only exhibit high photoluminescence quantum yields (PLQY), tunable molecular design, and facile synthesis but also demonstrate highly sensitive X-ray responsiveness [18,19,20]. Numerous researchers have explored and reported on their scintillation performance. For instance, Ma et al. utilized a solvent diffusion method to obtain high-quality (C_38_H_34_P_2_)[MnBr_4_] crystals [1], achieving quantum yields and photon yields of 95% and 80,000 photons MeV^−1^, respectively. Similarly, Lie et al. reported two examples of OIMnH crystals (Br-PrTPP)_2_[MnBr_4_] (Br-PrTPP = (3-bromopropyl) triphenylphosphonium) and (Br-BuTPP)_2_[MnBr_4_] (Br-BuTPP = (4-bromobutyl) triphenylphosphonium) [21]. The combination of high PLQY and low self-absorption in these halides endows them with exceptional scintillation capabilities, exhibiting photon yields exceeding 60,000 photons MeV^−1^. These examples demonstrate the potential of OIMnHs as excellent scintillators.

In recent years, studies have reported the correlation between the optical properties of OIMnHs and their structural configuration [22,23]. There is a direct relationship between the Mn···Mn distance and the PLQY. When the Mn···Mn distance is short, the electronic interaction and non-radiative energy transfer between adjacent [MnX_4_]^2−^ (X = Cl, Br) units lead to a decrease in the PLQY, which can be attributed to the manifestation of the concentration quenching effect. To address this problem, Mao et al. [24] demonstrated that bulky, rigid protonated cations can increase the Mn···Mn distance, thereby enhancing the PLQY of the compounds. Consequently, using larger cations to separate individual Mn polyhedral units serves as a viable strategy to reduce non-radiative electronic transitions, thereby improving the optical properties of the compounds. Numerous reports indicate that the radiative pathways of OIMnHs closely resemble those of photoluminescence pathways [25,26]. However, to date, there are still few studies on optimizing the Mn spacing through molecular design to simultaneously improve the efficiency of X-ray absorption and radiation transition. In particular, the mechanism of large steric cations with rigid skeletons in inhibiting non-radiative recombination has not been clarified.

In this study, we synthesized a 0-D organic-inorganic hybrid manganese halide compound using *N*,*N*,*N*-trimethyladamantan-1-aminium hydroxide in combination with MnBr_2_ via a solution method. The introduction of bulky cations effectively expanded the Mn···Mn distance, thereby reducing the concentration quenching effects from Mn atoms, leading to a quantum yield of up to 63.8%. Additionally, the compound exhibited excellent X-ray absorption capabilities, resulting in a photon yield of 44,300 photons MeV^−1^. This work provides a novel approach for the development of non-toxic, highly luminescent OIMnH scintillators, broadening the selection of materials for scintillation phosphors.

## 2. Results and Discussions

### 2.1. Crystal Structure Descriptions

**Figure 1:**
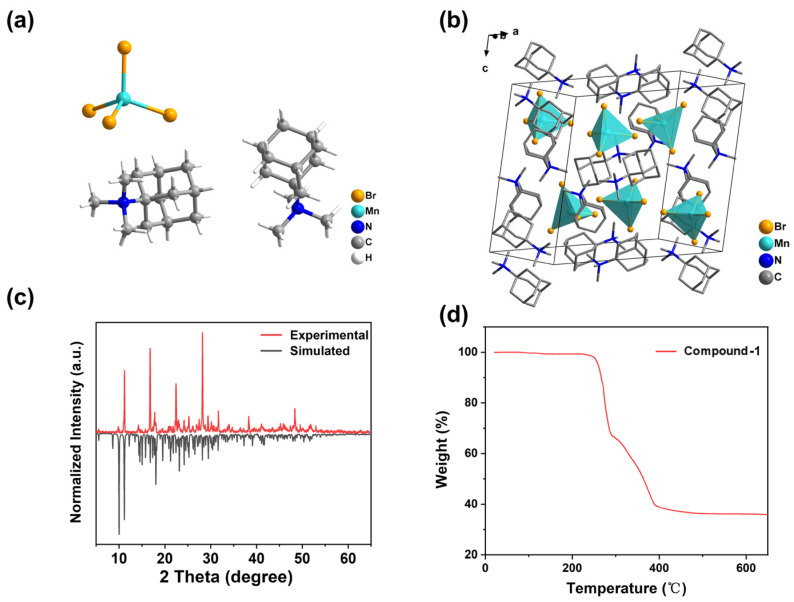
(**a**) Asymmetric structural unit diagram of compound **1**. (**b**) Packing crystal structures of compound **1**. (**c**) Powder X-ray diffraction (PXRD) patterns of compound **1** sample compared with crystal simulation patterns. (**d**) Thermogravimetric curves of compound **1**.

Crystals of (C_13_H_25_N)_2_[MnBr_4_] were successfully isolated via a simple solution evaporation method. X-ray single-crystal diffraction analysis revealed that the crystals belong to the monoclinic system, with space group *P*2_1_/c. The unit cell parameters were determined to be *a* = 16.0755(11) Å, *b* = 13.4085(7) Å, *c* = 14.6954(9) Å, *α* = *γ* = 90°, and *β* = 100.322(6)°. The detailed crystal structure and additional specifics are depicted in Table S1. As illustrated in Figure 1a, the asymmetric unit of compound **1** comprises two *N*,*N*,*N*-trimethyl-1-adamantanammonium cations and one [MnBr_4_]^2−^ anion unit. The Mn-Br bond lengths range from 2.5072(14) to 2.5307(13) Å, while the Br-Mn-Br bond angles vary from 106.73(5)° to 114.10(5)° (Table S2). The isolated [MnBr_4_]^2−^ tetrahedra are separated by the [C_13_H_25_N] organic cations, resulting in a characteristic 0-D structure (Figure 1b). The shortest Mn–Mn distance is measured at 9.034 Å (Figure S1), effectively mitigating spin-spin coupling between Mn ions in adjacent [MnBr_4_]^2−^ tetrahedral units [27,28]. Therefore, we speculate that the green emission from the crystal is primarily attributed to the Mn^2+^ ions. The powder X-ray diffraction (PXRD) data of the powdered sample were compared with those simulated from the single-crystal X-ray data, as shown in Figure 1c. The diffraction peaks of the obtained sample align well with the simulated one from the single-crystal X-ray data, and no additional impurity peaks were detected, indicating a high phase purity of the synthesized crystal. Additionally, thermogravimetric analysis (TGA) was conducted to assess the thermal stability of the compound, as illustrated in Figure 1d. The compound exhibits thermal decomposition at approximately 270 °C, confirming its excellent thermal stability.

### 2.2. The Luminescence Characteristics of Compound ***1***

**Figure 2:**
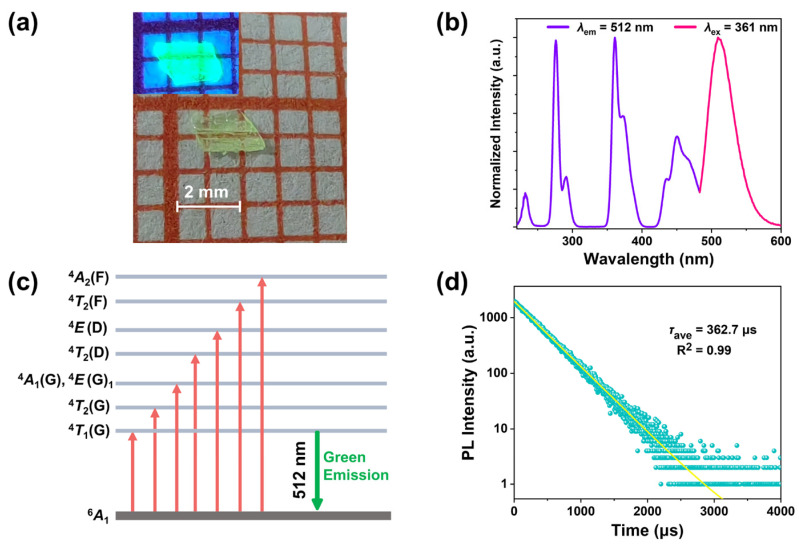
(**a**) Images of compound **1** crystals under the ambient light and the 365 nm UV light (size: 7 mm × 5 mm × 2 mm). (**b**) Normalized PLE (*λ*_em_ = 512 nm) and PL (*λ*_ex_ = 361 nm) spectra of compound **1** SCs. (**c**) Energy state splitting and optical transitions in Mn^2+^. (**d**) RT PL decay curves of **1** at 512 nm emission peak upon 362 nm excitation.

Further investigations were conducted to explore the optical properties of the compound. As illustrated in Figure 2a, the optical image of crystal **1** shows a crystal size of 2 mm × 1.5 mm × 1 mm, exhibiting green transparency under natural light. However, upon excitation with a 365 nm UV lamp, a bright green luminescence was observed. The excitation emission spectra were measured at room temperature, as shown in Figure 2b. The photoluminescence excitation (PLE) spectrum reveals three strong excitation peaks located at 276 nm, 361 nm, and 450 nm, which can be attributed to the transitions between the energy levels of ^4^A_2_(F)←^6^A_1_(S), ^4^E(D)←^6^A_1_(S), and ^4^T_2_(G)←^6^A_1_(S), respectively (Figure 2c). Photoluminescence (PL) spectra confirmed a broad green emission at 510 nm, with a full width at half maximum (FWHM) of 45 nm. The Stokes shift was measured to be 250 nm, indicating that the compound has an effective ability to minimize self-absorption [29]. As shown in Figure 2d, the PL decay curve of the compound was measured, and it was well-fitted using a single-exponential function for the decay lifetime (Equation (1)).

(1) $$I(T)=I_{0}\;\cdot \;e\;(-\frac{t}{\tau })$$

Fitting using Equation (1) yielded a PL lifetime of 362.7 μs for **1**. The single-exponential decay further confirms that the emission from Mn^2+^ in this compound arises from a singular radiative process. The long decay time further validates that the green emission originates from the transition of tetrahedrally coordinated Mn^2+^ from ^4^T_1_(G) to ^6^A_1_ [27]. The PLQY reached as high as 63.8% (Figure S2). Research indicates that there is a direct correlation between the Mn···Mn distance and the PLQY in manganese-based compounds [30]. As the Mn···Mn distance increases, the energy transfer efficiency between manganese ions is effectively reduced, suppressing non-radiative recombination. The introduction of rigid large cations expands the distance between Mn···Mn, thereby contributing to the high PLQY of **1**.

**Figure 3:**
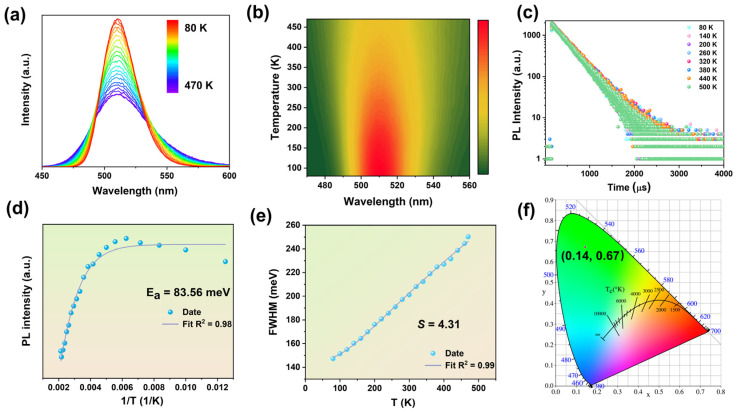
(**a**) PL spectra of compound **1** under heating conditions. (**b**) Temperature-dependent PL correlation plot of compound **1** under 361 nm excitation. (**c**) Time-resolved photoluminescence (TDPL) spectra of compound **1** under heating conditions. (**d**) PL intensities of compound **1** as a function of temperature under heating conditions. (**e**) FWHM intensities of compound **1** as a function of temperature under heating conditions. (**f**) CIE chromaticity diagram of compound **1**.

To elucidate the luminescent properties of **1**, temperature-dependent PL spectra were systematically investigated from 80 to 470 K. As depicted in Figure 3a, the compound exhibits a single-band emission across the entire temperature range, with no additional emission bands emerging upon thermal variation. This observation underscores the remarkable structural stability of **1**, as well as the absence of temperature-induced phase transitions or structural distortions. Upon increasing temperature, the PL emission intensity gradually decreases (Figure 3b), which can be attributed to thermally activated non-radiative recombination. As the vibrational energy levels rise at elevated temperatures, the enhanced coupling between the ground and excited states promotes non-radiative decay pathways, thereby diminishing the PL efficiency. Furthermore, time-resolved photoluminescence decay analysis reveals a gradual reduction in the PL lifetime with increasing temperature (Figure 3c), consistent with the enhanced non-radiative decay pathways. A single emission mechanism for compound **1** was also confirmed. Remarkably, the Commission Internationale de l’Éclairage (CIE) 1931 chromaticity coordinates of (0.14, 0.67) (Figure 3d) demonstrate a certain purity of green emission that closely matches the standard green locus.

To further explore the optical properties, we fitted a combined calculation of the integral PL intensity and FWHM versus temperature using the following equations [31]:

(2) $$I(T)=\frac{I_{0}}{1+Ae^{-\frac{E_{a}}{K_{b}T}}}$$

(3) $$\mathrm{FWHM}=2.36\sqrt{S}{\hbar \omega }_{\mathrm{phonon}}\sqrt{\coth\frac{{\hbar \omega }_{\mathrm{phonon}}}{2K_{B}T}}$$

where *I*(T) and *I*_0_ are the integrated emission intensities at different temperatures (T) and 0 K, respectively, and *K*_B_ is the Boltzmann constant. The FWHM in the equation represents the full width at half maximum at different temperatures, *ħω* phonon stands for the phonon vibration energy, and *S* denotes the Huang–Rhys factor. The thermal (exciton binding) activation energy (*E*_a_) reflects the material’s ability to overcome thermal quenching effects. The fitted *E*_a_ value during the warming process is 83.5 meV (Figure 3e), much larger than the thermal ionization energy at room temperature (26 meV) [32], and the large *E*_a_ value can effectively promote the radiative composite probability of excitons, thus effectively improving the PL emission efficiency. The Huang–Rhys factor (*S*) is usually used to assess the strength of the electron-phonon coupling, related to the Jahn-Teller distortion [33]. The *S*-value was fitted to 4.31 meV (Figure 3f), indicating weak electron–phonon coupling in the structure of the compound, similar to the currently reported Mn-based halides [12].

**Figure 4:**
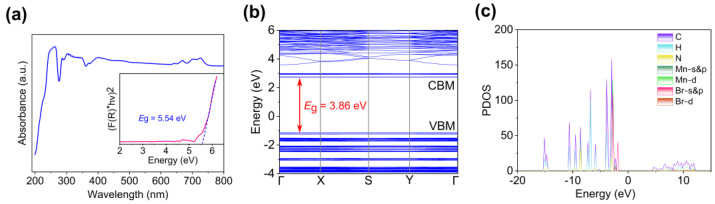
(**a**) UV–vis spectra of compound **1**. (**b**) Band structure and (**c**) DOS diagram of compound **1**.

Subsequently, the optical properties of the crystal were investigated using UV–Vis diffuse reflectance absorption spectroscopy. A distinct absorption edge was observed in the range of 200 nm to 480 nm, corresponding to transitions from the ground state to various excited states within the compound [34]. The optical band gap value can be calculated using the following equation:

[F(R∞)*hv*]*_n_* = A(*hv* − *E*_g_) (4)

where F(R∞) is the Kubelka–Munk (K–M) function, *hν* is the photon energy, A is a proportionality constant, *E*_g_ is the band gap, and *n* = 2/0.5 for the direct/indirect semiconductor. It is concluded that compound **1** exhibits characteristics of a direct band gap structure, with an optical band gap value of approximately 5.54 eV (Figure 4a). From the UV–visible spectrum and PLE spectrum of compound **1**, we found that there are excitation bands at 267 nm, 361 nm, and 450 nm. Figure S4 shows the emission peaks of different excitation bands, and the emission peaks do not change, which is attributed to the d-d transition of the tetrahedral coordinated Mn^2+^ center in the [MnBr_4_] unit. In addition, we calculated the electronic band structure and density of states (DOS) of compound **1** using density functional theory (DFT). As observed in Figure 4b, the band fluctuations in the Brillouin zone are relatively flat, exhibiting localized characteristics. This behavior is attributed to the absence of orbital overlap and the strong quantum confinement effects caused by the isolated [MnBr_4_] units within the 0-D structure. The minimum value of the conduction band (CBM) and the maximum value of the valence band (VBM) are located at the same position, with the calculated direct band gap estimated to be approximately 3.86 eV (Figure 4b). This value is lower than the experimental value, which is attributed to the slight underestimation of the band gap by the PBE functional in DFT [35,36]. The projected density of states further illustrates the contribution of atomic orbitals to the spectral bands (Figure 4c). The results indicate that the VBM is primarily derived from the 4p orbitals of Br and the 3d orbitals of Mn, while the CBM is mainly contributed by the orbitals of the organic components. These findings suggest that the band absorption in compound **1** is primarily attributed to charge transfer from the inorganic [MnBr_4_] tetrahedra to its organic moieties.

### 2.3. The Scintillation Characteristics of Compound ***1***

**Figure 5:**
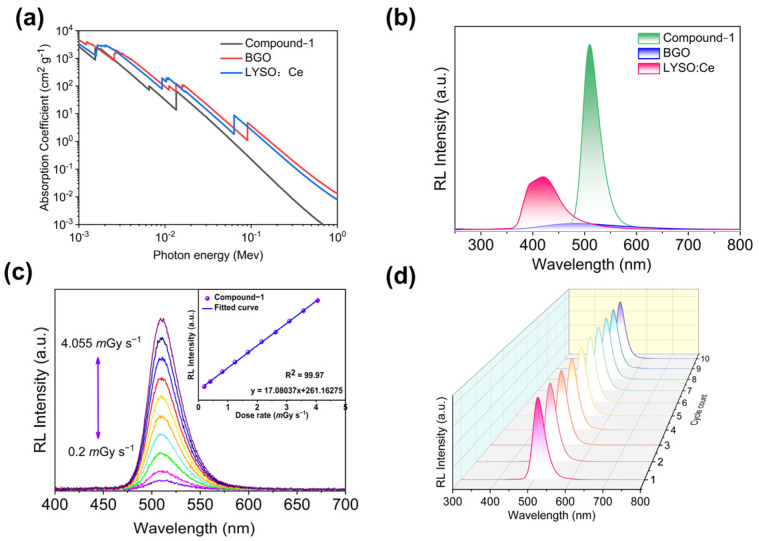
(**a**) Absorption coefficients of compound **1** and LuAG: Ce, BGO as a function of photon energy from 0.001 to 1 Mev. (**b**) The RL spectra of compound **1**, BGO, and LuAG: Ce under the excitation of X-ray (50 kV, 100 μA). (**c**) RL emission spectra of compound **1** at different X-ray dose rates. Insert: integrated scintillation intensity as a linear function of the X-ray dose rate. (**d**) RL emission spectra of compound 1 under irradiation of X-ray with a dose rate of 177.5 *m*Gy_air_ s^−1^ for 10 cycles.

Based on the excellent optical properties of compound **1**, we further investigated its scintillation performance. The attenuation ability of X-rays is one of the important parameters for evaluating scintillator performance. As shown in Figure 5a, we plotted the variation of the absorption coefficients of compound **1** alongside two typical commercial scintillators (BGO and LYSO: Ce) as a function of photon energy. The results indicate that the absorption of X-rays by compound **1** is slightly lower than that of the commercial scintillators, which may be attributed to the lower density of the organic–inorganic hybrid material (C_13_H_25_N)_2_[MnBr_4_] and the presence of fewer heavy elements. Using the commercial scintillators BGO and LYSO: Ce as references, we tested the radioluminescence (RL) spectrum of compound **1** under X-ray irradiation at the same dose rate of 177.5 *m*Gy_air_ s^−1^ (Figure 5b). It was observed that the RL spectrum intensity of compound **1** is significantly higher than that of the commercial scintillators. Furthermore, the RL emission spectrum of the compound is similar to its PL emission, indicating that it follows the same radiative recombination pathway under both ultraviolet and X-ray excitation. By comparing the integrated RL emission spectrum areas of BGO (~8000 photons/MeV) [37], LYSO: Ce (~33,000 photons/MeV) [38] (Equation (S1)), and compound **1**, the average scintillation light yield of compound **1** was calculated to be approximately 44,300 photons/MeV (Figure 5b). To investigate the X-ray detection sensitivity of the crystal, the RL spectra were recorded under different X-ray dose rates (Figure 5c). Compound **1** exhibited consistent RL emission, showing a monotonic increase in radiation response as the dose rate increased from 0.2 *m*Gy_air_ s^−1^ to 4.055 *m*Gy_air_ s^−1^, with excellent linear response. This indicates that the emission mechanism remains unchanged. Additionally, we tested the RL stability of compound **1** over 10 cycles (Figure 5d and Figure S5). After ten cycles, the emission spectrum of the compound remained unchanged, demonstrating that it possesses good radiation stability. These results indicate that (C_13_H_25_N)_2_[MnBr_4_] has significant potential for the fabrication of high-performance X-ray detectors.

## 3. Materials and Methods

Manganese(II) bromide tetrahydrate (MnBr_2_·4H_2_O, Maclin, 99%, Energy Chemical, Shanghai, China), *N*,*N*,*N*-trimethyladamantan-1-aminium hydroxide (C_13_H_24_NOH, Maclin, 99%, Energy Chemical, Shanghai, China), hydrogen bromide (HBr, Maclin, 99%, Energy Chemical, Shanghai, China), and deionized water (H_2_O, Maclin, 99%) were used. All chemicals were purchased from Energy Chemical Company, Shanghai, China, without further purification.

**Preparation of compound 1.** Compound of (C_13_H_25_N)_2_[MnBr_4_] was obtained by the slow evaporation solvent method. The mixture of 3 mmol (698 mg) MnBr_2_·4H_2_O and 20 mL of HBr (40% aq) was added to a small beaker, stirred, and reacted to obtain a clear solution. Then, 3 mmol (634 mg) trimethyladamantan-1-aminium hydroxide was added reaction system, and then 20 mL of deionized water was added and stirred thoroughly until there was no obvious precipitation. The green transparent crystal of **1** was obtained by slowly evaporating at room temperature overnight, Yield, 1.26 g, 55.0% based on Mn. The element analysis for (C_13_H_25_N)_2_[MnBr_4_] Anal. (%) calc.: C 40.81, H 6.59, N 3.66. Found (%): C 40.66, H 6.90, N 3.57.

**Characterizations**. Single-crystal X-ray diffraction (SCXRD) data were collected on a XtaLAB Synergy R, HyPix instrument (Rigaku, Tokyo, Japan) using graphite-monochromated Mo-Kα radiation (*λ* = 0.71073 Å) at 296 K. The structure was solved using SHELXT methods with the Olex2 program [39,40], and all non-hydrogen atoms were refined anisotropically by the least-squares technique on weighted F2 using SHELXL [41]. Powder X-ray diffraction (PXRD) data were recorded on a Ultima IV diffractometer (Rigaku, Tokyo, Japan) using graphite-monochromated Cu-K*α* radiation (*λ* = 1.5406 Å). Thermogravimetric analysis (TGA) was conducted using a STA 449F3 thermal analyzer (NETZSCH, Selb, Germany) and the sample was heated in the temperature range of 300–800 °C under a nitrogen atmosphere. The diffuse reflectance spectra of the powder samples were measured at room temperature in the wavelength range of 200–800 nm using a 2600 UV/Vis spectrometer (Shimadzu, Kyoto, Japan). Photoluminescence (PL) and photoluminescence excitation (PLE) spectra and photoluminescence quantum yield (PLQY) were obtained using a PL spectrometer (FLS980; Edinburgh Instruments, Scotland, UK). Temperature-dependent PL spectra and PL decay curves were measured using an FLS980 spectrometer (Edinburgh, Scotland, UK). The RL spectra and RL stability cycle data were acquired using an X-XILS-P70V-5 variable-temperature X-ray luminescence spectrometer system (Jiangxi Xinwei Instrument Co., Ltd., Jiujiang, Jiangxi, China).

CCDC NO. 2444825 (for compound **1**), contains the supplementary crystallographic data for this paper. These data can be obtained free of charge from The Cambridge Crystallographic Data Centre via www.ccdc.cam.ac.uk/data_request/cif.

**Theoretical Band Calculation.**

All calculations using density functional theory (DFT) were carried out using the Vienna Ab initio simulation package (VASP) [42]. The band structure calculations were performed based on the optimized geometric configurations. The special points along high-symmetry paths, such as Gamma, X, M, and R, were automatically selected using VASPkit. The valence band maximum (VBM) and conduction band minimum (CBM) were determined through wave function analysis conducted with VASPkit, and the results were visualized using VESTA. The density of states (DOS) calculations were performed using the same k-point grid as that employed for the band structure calculations.

## 4. Conclusions

In summary, we synthesized a novel organic–inorganic hybrid manganese halide crystal using a solution-based method. Under 365 nm excitation, it exhibited bright green luminescence. The presence of bulky organic cations increased the Mn···Mn distances, thereby mitigating concentration quenching and resulting in a high PLQY of up to 63.8%. The 0-D structure, along with the large Stokes shift, decreased the self-absorption effect, achieving a light yield of 44,300 photons/MeV. This work provides new insights for the preparation of bright, non-toxic OIMnHs scintillators and broadens the selection of materials for scintillation phosphors.

## Data Availability

The original contributions presented in the study are included in the article/Supplementary Materials; further inquiries can be directed to the corresponding authors.

## Supplementary Materials

The following supporting information can be downloaded at: https://www.mdpi.com/article/10.3390/molecules30102157/s1, Figure S1: The adjacent Mn-Mn distances (Å) in (C_13_H_25_N)_2_MnBr_4_, labeled with green lines.; Figure S2: The PLQY spectrum of compound **1**; Figure S3: Temperature-dependent PL intensity of compound **1**; Figure S4: PL spectra of **1** were measured at different excitation wavelengths; Figure S5: The RL response of compound 1 under irradiation of X-ray with a dose rate of 177.5 *m*Gy_air_ s^−1^ for 10 cycles; Table S1: Crystallographic data and structural refinement details for (C_13_H_25_N)_2_MnBr_4_; Table S2: Table of selected bond lengths (Å) and bond angles (°) for compound of **1**.
