# Supplementary material for: Highly Luminescent and Scintillating Hybrid Halide of (C13H25N)2[MnBr4] Enabled by Rigid Cation
Source: Molecules. 2025 May 14;30(10):2157. doi: 10.3390/molecules30102157 (PMC12114584; doi:10.3390/molecules30102157)
Supplement: Supplementary file 1 [file molecules-30-02157-s001.zip › molecules-3633224-supplementary.pdf]

Supporting Information

Highly Luminescent and Scintillating Hybrid Halide of $(C_{13}H_{25}N)_2[MnBr_4]$ Enabled by Rigid Cation

Renfu Li, Lulu Jiang, Qinghua Zou, Jianlong Bai, Lingkun Wu, Jianrong Li * and Jinsheng Liao *

Jiangxi Provincial Key Laboratory of Functional Crystalline Materials Chemistry, Jiangxi University of Science and Technology, Ganzhou 341000, China; lirenfu@fjirsm.ac.cn (R.L.); baekrrlulu@163.com (L.J.); zouqinghua1126@163.com (Q.Z.); baijianlong666@gmail.com (J.B.); wlk492599141@163.com (L.W.)

* Correspondence: jrli@fjirsm.ac.cn (J.L.); jsliao1209@126.com (J.L.)

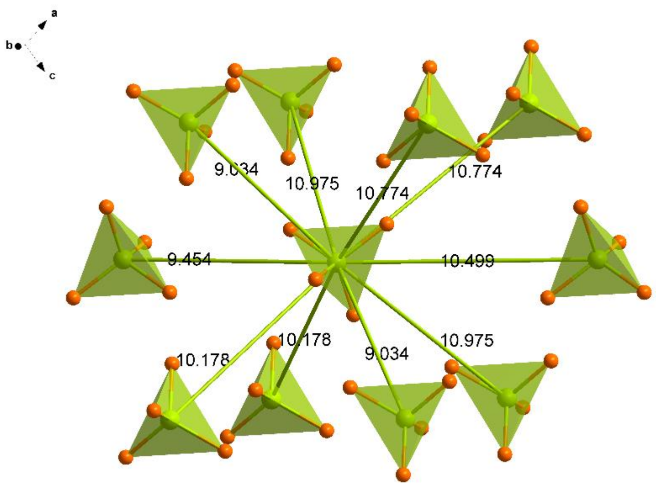

Figure S1. The adjacent Mn–Mn distances (Å) in $(C_{13}H_{25}N)_2MnBr_4$, labeled with green lines.

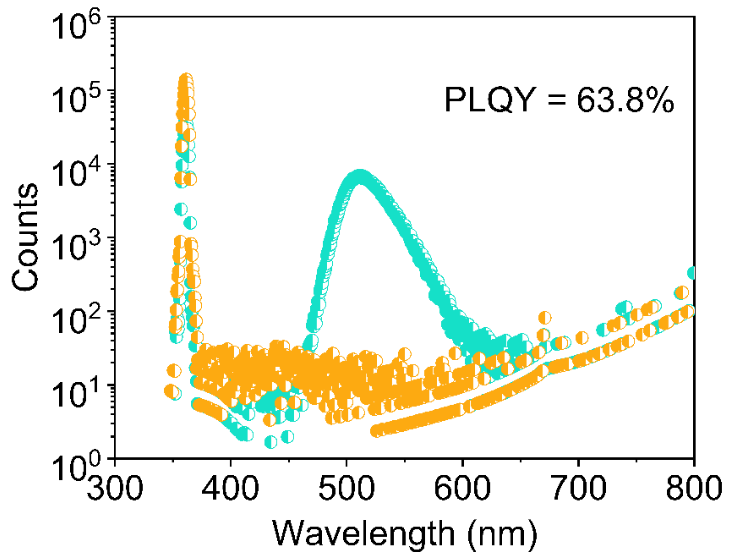

Figure S2. The PLQY spectrum of compound **1**.

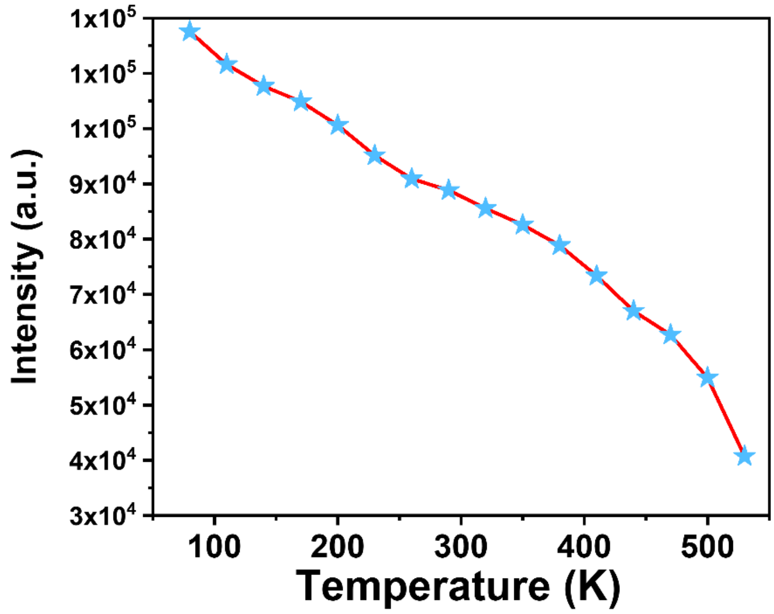

Figure S3. Temperature-dependent PL intensity of compound **1**.

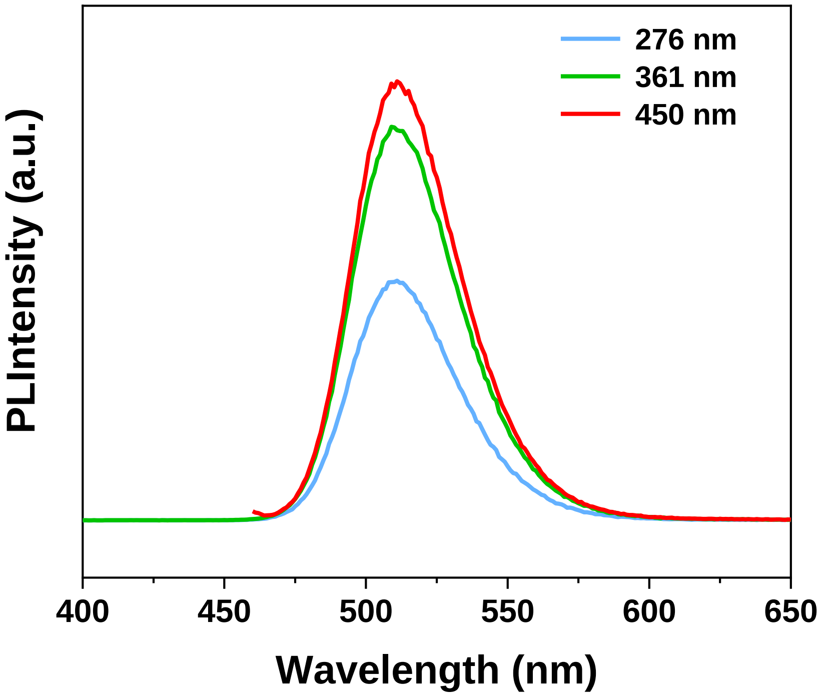

Figure S4. PL spectra of **1** measured at different excitation wavelengths.

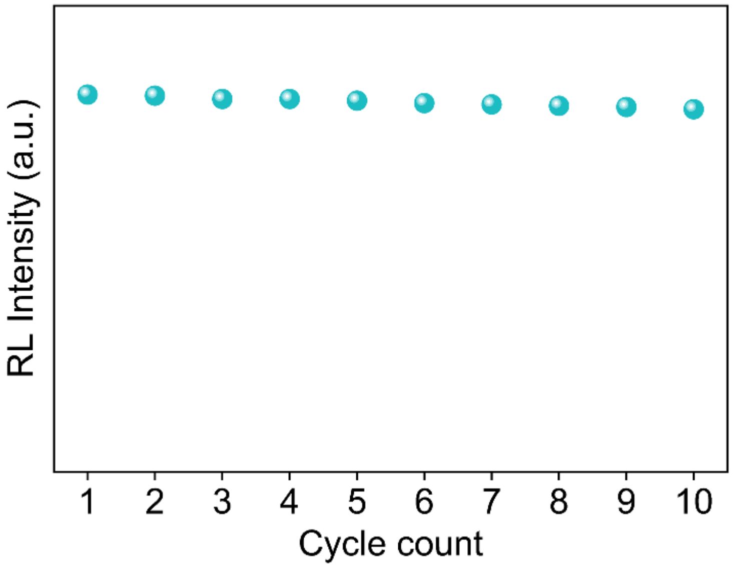

Figure S5. The RL response of compound **1** under irradiation of X-ray with a dose rate of 177.5 *m*Gy_{air} s^{-1} for 10 cycles.

Table S1. Crystallographic data and structural refinement details for $(C_{13}H_{25}N)_2MnBr_4$.

**Parameter**	$\mathbf{(C_{13}H_{25}N)_2MnBr_4}$
T / K	285.0(4)
Formula	$C_{26}H_{48}Br_4MnN_2$
Formula weight	763.24
Crystal system	Monoclinic
Space group	$P2_1/c$
a /Å	16.0755(11)
b /Å	13.4085(7)
c /Å	14.6954(9)
α /°	90
β /°	100.322(6)
γ /°	90
V (Å^3)	3116.3(3)
Z	4
λ /Å	0.71073
F(000)	1532.0
2θ	3.982-58.324
R_1[a]/wR_2[b] [I>=2σ (I)]	0.0740-0.1251
R_1[a]/wR_2[b] (all data)	0.1460-0.1481
Reflections collected	22399
CCDC	2444825

[a] $R_1 = \Sigma||F_o|-|F_c||/|F_o|$; [b] $wR_2 = [\Sigma w(F_o^2-F_c^2)^2]/\Sigma w(F_o^2)^2]^{1/2}$.

Table S2. Table of selected bond lengths (Å) and bond angles (°) for compound of **1**.

Br1–Mn1	2.5155(13)	C11–C12	1.521(11)
Br2–Mn1	2.5072(14)	C12–C13	1.526(11)
Br3–Mn1	2.5307(13)	N2–C14	1.497(10)
Br4–Mn1	2.5216(14)	N2–C15	1.505(10)
N1–C1	1.516(9)	N2–C17	1.490(10)
N1–C2	1.505(9)	N2–C18	1.577(9)
N1–C3	1.505(9)	C16–C18	1.536(10)
N1–C5	1.571(9)	C16–C19	1.525(10)
C4–C5	1.544(9)	C18–C21	1.534(10)
C4–C7	1.524(11)	C18–C26	1.531(10)
C5–C6	1.537(9)	C19–C20	1.517(12)
C5–C8	1.530(10)	C19–C25	1.522(11)
C6–C10	1.527(11)	C20–C22	1.516(14)
C7–C9	1.529(11)	C21–C22	1.534(13)
C7–C11	1.521(12)	C22–C23	1.504(14)
C8–C12	1.542(11)	C23–C24	1.521(13)
C9–C10	1.526(12)	C24–C25	1.502(12)
C10–C13	1.523(11)	C24–C26	1.543(11)
Br1–Mn1–Br3	106.73(5)	C4–C5–N1	109.4(6)
Br1–Mn1–Br4	108.42(5)	C6–C5–N1	110.4(5)
Br2–Mn1–Br1	114.10(5)	C6–C5–C4	109.1(6)
Br2–Mn1–Br3	108.58(5)	C8–C5–N1	109.9(5)
Br2–Mn1–Br4	110.65(5)	C8–C5–C4	108.9(6)
Br4–Mn1–Br3	108.15(5)	C8–C5–C6	109.2(6)
C1–N1–C5	111.3(5)	C9–C10–C6	110.9(7)
C2–N1–C1	106.1(6)	C13–C10–C6	109.6(7)
C2–N1–C3	108.1(6)	C13–C10–C9	109.2(7)
C2–N1–C5	112.5(5)	C4–C7–C9	110.6(7)
C3–N1–C1	106.8(6)	C11–C7–C4	110.7(7)
C3–N1–C5	111.6(6)	C11–C7–C9	108.5(7)
C11–C12–C8	109.1(7)	C5–C8–C12	108.9(6)
C11–C12–C13	110.0(7)	C7–C4–C5	109.1(6)
C13–C12–C8	111.1(7)	C12–C11–C7	108.8(7)
C10–C13–C12	108.1(6)	C14–N2–C15	106.2(8)
C19–C16–C18	110.0(6)	C14–N2–C18	111.4(5)
C16–C18–N2	109.8(6)	C15–N2–C18	112.5(6)

C21–C18–N2	111.1(6)	C17–N2–C14	107.1(7)
C21–C18–C16	107.9(6)	C17–N2–C15	107.7(7)
C26–C18–N2	110.5(6)	C17–N2–C18	111.6(7)
C26–C18–C16	108.7(6)	C23–C22–C21	109.7(9)
C26–C18–C21	108.9(7)	C23–C22–H22	109.4
C20–C19–C16	109.9(7)	C22–C23–C24	109.3(8)
C20–C19–C25	109.5(7)	C23–C24–C26	108.8(8)
C25–C19–C16	109.6(7)	C25–C24–C23	110.3(8)
C22–C20–C19	109.3(7)	C24–C25–C19	109.4(7)
C22–C21–C18	110.5(7)	C25–C24–C26	110.2(7)
C18–C26–C24	109.6(6)	C23–C22–C20	110.9(9)
C20–C22–C21	108.1(8)		

Equation S1： The light yield of $(C_{13}H_{25}N)_2MnBr_4$ was estimated by comparing the integral areas of its radioluminescence (RL) emission spectrum with those of the reference standards, the commercial scintillators BGO (8000 photons/MeV) and LYSO: Ce. To minimize experimental errors, the test samples were fabricated into test blocks of the same dimensions as the standard samples (5 mm × 5 mm × 1 mm). All spectral measurements were conducted under the same voltage and current conditions (50 kV, 100 μA), with the X-ray source positioned 1 cm from the sample, and the estimation was subsequently performed using the following formula:

$$\frac{\mathrm{L}Y_{\mathrm{sample}}}{\mathrm{L}Y_{\mathrm{BGO\ /\ LYSO:\ Ce}}}=\frac{R_{\mathrm{sample}}}{R_{\mathrm{BGO\ /\ LYSO:\ Ce}}}\times\frac{\int I_{\mathrm{BGO\ /\ LYSO:\ Ce}}(\lambda)/\mathrm{S}(\lambda)\int I_{\mathrm{BGO\ /\ LYSO:\ Ce}}(\lambda)\mathrm{d}\lambda}{\int I_{\mathrm{sample}}(\lambda)/\mathrm{S}(\lambda)\int I_{\mathrm{sample}}(\lambda)\mathrm{d}\lambda}$$

where R is defined as the X-ray deposited energy percentage of scintillators, $I(\lambda)$ is the radioluminescence spectrum at different wavelengths, and $S(\lambda)$ represents the detection efficiency at different irradiation area, respectively.
